# Time trends (2001–2019) and sex differences in incidence and in-hospital mortality after lower extremity amputations among patients with type 1 diabetes in Spain

**DOI:** 10.1186/s12933-022-01502-y

**Published:** 2022-05-03

**Authors:** Ana Lopez-de-Andres, Rodrigo Jimenez-Garcia, Valentín Hernández-Barrera, Javier de-Miguel-Diez, José M. de-Miguel-Yanes, Ricardo Omaña-Palanco, David Carabantes-Alarcon

**Affiliations:** 1 Department of Public Health & Maternal and Child Health. Faculty of Medicine, Universidad Complutense de Madrid, Madrid, Spain; 2 Preventive Medicine and Public Health Teaching and Research Unit. Health Sciences Faculty. Rey Juan Carlos University. Alcorcón, Madrid, Spain; 3 Respiratory Care Department, Hospital General Universitario Gregorio Marañón, Universidad Complutense de Madrid, Instituto de Investigación Sanitaria Gregorio Marañón (IiSGM), Madrid, Spain; 4 Internal Medicine Department. Hospital General, Universitario Gregorio MarañónUniversidad Complutense de MadridInstituto de Investigación Sanitaria Gregorio Marañón (IiSGM), Madrid, Spain

**Keywords:** Amputation: Lower extremity, Diabetes, Hospitalization, Mortality, Sex differences

## Abstract

**Background:**

We examined trends in incidence (2001–2019), clinical characteristics, and in-hospital outcomes following major and minor lower extremity amputations (LEAs) among type 1 diabetes mellitus (T1DM) patients in Spain and attempted to identify sex differences.

**Methods:**

Retrospective cohort study using data from the Spanish National Hospital Discharge Database. We estimated the incidence of the LEA procedure stratified by type of LEA. Joinpoint regression was used to estimate incidence trends, and logistic regression was used to estimate factors associated with in-hospital mortality (IHM).

**Results:**

LEA was coded in 6011 patients with T1DM (66.4% minor and 33.6% major). The incidence of minor LEA decreased by 9.55% per year from 2001 to 2009 and then increased by 1.50% per year, although not significantly, through 2019. The incidence of major LEA decreased by 13.39% per year from 2001 to 2010 and then remained stable through 2019. However, incidence increased in men (26.53% per year), although not significantly, from 2017 to 2019. The adjusted incidence of minor and major LEA was higher in men than in women (IRR 3.01 [95% CI 2.64–3.36] and IRR 1.85 [95% CI 1.31–2.38], respectively). Over the entire period, for those who underwent a minor LEA, the IHM was 1.58% (2.28% for females and 1.36% for males; p = 0.045) and for a major LEA the IHM was 8.57% (10.52% for females and 7.59% for males; p = 0.025).

IHM after minor and major LEA increased with age and the presence of comorbid conditions such as peripheral arterial disease, ischemic heart disease or chronic kidney disease. Female sex was associated with a higher IHM after major LEA (OR 1.37 [95% CI 1.01–1.84]).

**Conclusions:**

Our data show a decrease in incidence rates for minor and major LEA in men and women with T1DM and a slight, albeit insignificant, increase in major LEA in men with T1DM in the last two years of the study. The incidence of minor and major LEA was higher in men than in women. Female sex is a predictor of IHM in patients with T1DM following major LEA.

**Supplementary Information:**

The online version contains supplementary material available at 10.1186/s12933-022-01502-y.

## Background

Lower extremity amputation (LEA) is a major complication of diabetes and is associated with low quality of life and higher risk of short-term mortality [1]. People with type 1 diabetes mellitus (T1DM) have a higher risk of LEA [2]. A recent study showed a 40-fold greater risk of amputation in patients with T1DM than in the general population [3]. The risk of LEA in patients with T1DM is a result of the combination of several conditions whose key contributing factors include ageing, male sex, cardiovascular comorbidities, hyperglycaemia, hypertension, and hyperlipidaemia [2, 4].

Rates of LEA among adults with diabetes are an important index of comprehensive diabetes care. Population-based studies in Spain showed that the number of T1DM-related LEAs decreased between 2001 and 2008 and then remained stable through 2012 [5, 6]. A Swedish study also reported a decrease in the frequency of amputations in patients with T1DM between 2017 and 2019 [4]. Similar findings have been reported in Denmark [7]. However, a study performed in the United States found an increase in diabetes-related LEAs, predominantly among younger patients (aged 18–44 years) and middle-aged patients (45–64 years) [8].

Sex differences may play an active role in incidence and outcomes among patients with diabetes following LEA. Several studies indicate that male sex is associated with a higher incidence rate than female sex [5, 6, 9], although female sex is an independent predictor of surgical site infection and in-hospital mortality (IHM) following LEA [6, 10]. Data regarding the results of hospitalization following LEA among men and women with T1DM are scarce.

Reliable epidemiological data on trends related to LEA, sex differences, and associated mortality are useful for assessing whether the quality of care for people with T1DM has improved and for informing health policy makers and healthcare providers of where additional resources may be required to fill the care gaps. Therefore, we used administrative data from an entire country over an 18-year period to examine trends in the incidence, clinical characteristics, and in-hospital outcomes of LEA procedures (major and minor) among patients hospitalized with T1DM. We also attempted to identify possible sex differences. Furthermore, we investigated the variables associated with IHM according to the type of LEA.

## Methods

### Study design, setting, and study population

To achieve the objectives set, a retrospective cohort study was carried out based on hospital discharge reports collected through the Spanish Hospital Discharge Records Database of the Spanish National Health System (RAE-CMBD, *Registro de Actividad de Atención Especializada. Conjunto Mínimo Básico de Datos*, Registry of Specialized Health Care Activities. Minimum Basic Data Set) for the period running from 1 January 2001 to 31 December 2019. The discharge records are coded based on the International Classification of Diseases, 9th Revision, Clinical Modification (ICD-9-CM) from 2001 to 2015 and the 10^th^ Revision (ICD-10) from 2016 until the present. Detailed information on RAE-CMBD are available online [11]. For purposes of the study, we excluded data from the year 2016, because this was the year the RAE-CMBD began the conversion from ICD-9 to ICD-10 and, according to the Ministry of Health, some degree of under-coding may be present [12].

The study population included patients with T1DM aged ≥ 18 years with a procedure code for LEA in their discharge records. The ICD-9-CM and ICD-10 codes used to identify the study population are shown in Additional file 1: Table S1. We defined as a minor amputation any LEA distal to the ankle joint and as a major amputation any LEA through or proximal to the ankle joint, as described in a previous study [6].

We excluded patients with a diagnosis code of type 2 diabetes mellitus (T2DM) and all those with traumatic LEAs based on any lower extremity trauma-related code or diagnosis or procedure position (Additional file 1: Table S1).

For patients with multiple LEAs during their stay, only the higher-level LEAs was used in the data analysis.

### Study variables

The main study variables were the incidence of major and minor LEAs, clinical characteristics, length of hospital stay (LOHS), and IHM.

Incidence rates were calculated based on the Spanish population with diabetes mellitus grouped by age and sex according to the National Health Surveys conducted for the years 2001/2002, 2003/2004, 2006/2007, 2009/2010, 2011/2012, 2014/2015, and 2016/2017 and based on data from the Di@bet.es Study, which estimated the prevalence of diabetes in the Spanish population [13, 14]. Diabetic populations for the missing years (2005, 2008, 2013, and 2018) were estimated assuming that the growth rate was the same throughout the period.

The patient-level variables analysed included age and clinical condition present at admission or diagnosed during hospitalization. The clinical conditions analysed included those of the Charlson Comorbidity Index (CCI) calculated based on ICD-9 and ICD-10 codes, as described elsewhere [15]. The CCI was analysed as a continuous variable, divided into three categories (0, 1, and ≥ 2) and for each of the following specific conditions included in the index (ischemic heart disease, chronic kidney disease, stroke, heart failure, liver disease, dementia, chronic pulmonary disease, connective tissue disorder, peptic ulcer, cancer or metastatic cancer and, human immunodeficiency virus).

Furthermore, we specifically described and analysed a series of diseases owing to their high prevalence and importance in T1DM patients, as follows: peripheral arterial disease (PAD), infection, peripheral neuropathy, hypertension, and lipid metabolism disorders. (Additional file 1: Table S1). For the first three conditions ICD codes used were based in the recommendations of Lin et al. [16].

### Statistical analysis

The period from 2001 to 2019 was divided into six two-year periods: 2001–2003, 2004–2006, 2007–2009, 2010–2012, 2013–2015, and 2017–2019.

Descriptive statistics for continuous variables were reported as mean and standard deviation (SD). We conducted the skewness and kurtosis normality tests (Stata command *“sktest”*) for CCI and LOHS, finding that these variables are not normally distributed, so for these two variables medians with inter quartile range (IQR) are provided.

Categorical variables were reported as frequency and percentage. We performed this statistical analysis by stratifying LEAs as major and minor.

Continuous variables were compared using the t test (means) or Wilcoxon rank sum test (medians). Categorical variables were compared using the chi-square test.

Incidence was analysed using Poisson regression models adjusted for age. Incidence rate ratios (IRR) were reported with their 95% confidence intervals (95% CI).

We used log joinpoint regression to identify the period in which trends changes in minor and major LEA rates occurred for each year, as well as to estimate the annual percentage of change in each of the periods delimited by the points of change. The analysis started with the minimum number of joinpoints and tested whether the inclusion of one or more joinpoints was statistically significant [17]. In the final model, each joinpoint indicated a significant trend change, and the annual percentage of change was obtained in each of the segments delimited by the joinpoints using the weighted least squares technique. Joinpoint Regression Program, version 4.0.4, was used for the analysis.

We conducted multivariable logistic regression analysis to identify which demographic and clinical conditions present on admission were independently associated with IHM. We constructed models separately for minor LEA and major LEA. The variables included in these models were age, sex, year of admission and all those clinical conditions that showed a significant association with the IHM in the bivariate analysis.

The statistical analysis was conducted using Stata version 14 (Stata, College Station, Texas, USA), and significance was set at p < 0.05 (two-sided).

### Ethical aspects

The RAE-CMBD is owned by the Spanish Ministry of Health and can be accessed freely upon request [18]. Given the characteristics of this registry, which is anonymous, and according to Spanish legislation, ethics committee approval was not required.

## Results

A total of 6,011 non-traumatic amputations (3,992 minor and 2,019 major, ratio 1.97) were identified in patients aged ≥ 18 years with T1DM in Spain between 2001 to 2019. The proportion of women was 27.31% (1,642).

### Trends in incidence, clinical characteristics, and hospital outcomes for patients who underwent a minor LEA procedure

**Fig. 1:**
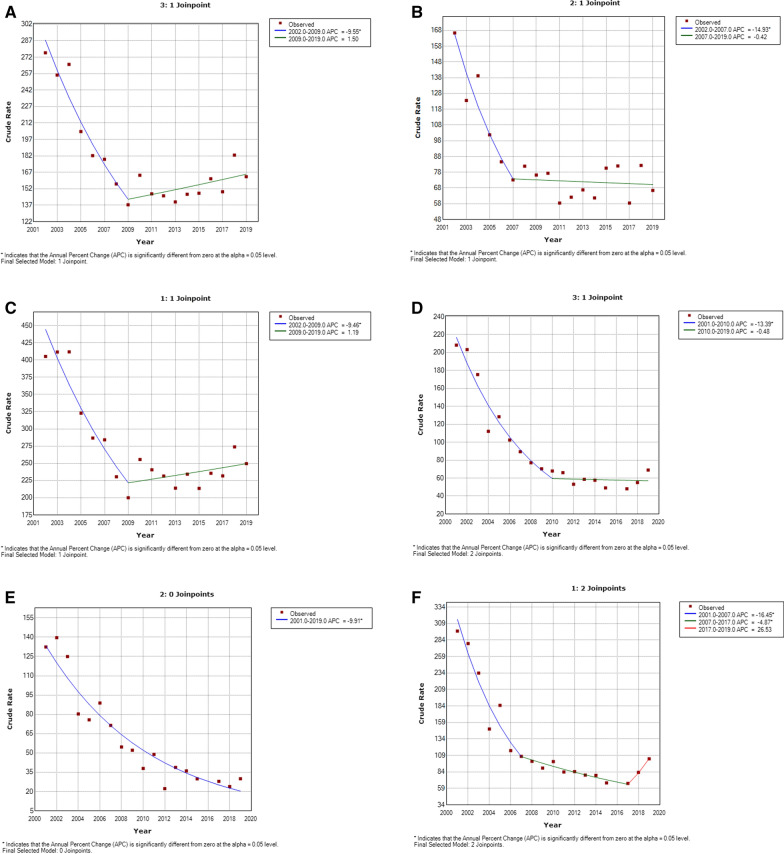
Joinpoint analysis of annual LEAS in patients with type 1 diabetes in Spain. **A** Joinpoint analysis of annual minor LEAs in women with T1DM in Spain, 2001–2019. **B** Joinpoint analysis of annual minor LEAs in men with T1DM in Spain, 2001–2019. **C** Joinpoint analysis of annual major LEAs in patients with T1DM in Spain, 2001–2019. **D** Joinpoint analysis of annual major LEAs in women with T1DM in Spain, 2001–2019. **E** Joinpoint analysis of annual major LEAs in men with T1DM in Spain, 2001–2019. APC: Annual percent change (based on rates that were sex and aged-adjusted using the Spanish National Statistics Institute Census projections) calculated using joinpoint regression analysis. ˆAPC is significantly different from zero (two-side P < 0.05)

According to the results of the joinpoint analysis, we found that the incidence of sex- and age-adjusted minor LEA procedures in patients with T1DM decreased by 9.55% per year from 2001 to 2009. From 2010 to 2019, they increased by 1.50% per year, although not significantly (Fig. 1A). According to sex, we found that in women with T1DM, the incidence rate decreased by 14.93% per year from 2001 to 2007 and remained stable thereafter (Fig. 1B). Incidence decreased significantly in men by 9.46% per year from 2001 to 2009, with no change from 2009 to 2019 (Fig. 1C).

As can been seen in Table 1, in patients who underwent minor LEAs, a significant increase was observed over time in the prevalence of men (70.94% in 2001/3 vs. 80.13% in 2017/9; p < 0.001).

**Table 1:** Hospital discharges after minor and major non-traumatic lower extremity amputations among T1DM patients (Spain, 2001–2019)

		2001–03	2004–06	2007–09	2010–12	2013–15	2017–19	Total	Trend
MINOR	n	826	619	547	576	634	790	3992	
	Male, n (%)	586 (70.94)	472 (76.25)	405 (74.04)	449 (77.95)	480 (75.71)	633 (80.13)	3025 (75.78)	0.013
	Female, n (%)	240 (29.06)	147 (23.75)	142 (25.96)	127 (22.05)	154 (24.29)	157 (19.87)	967 (24.22)	< 0.001
	Age, Mean (SD)	62.38 (13.29)	58.05 (14.98)	55.63 (14.01)	54.17 (12.8)	52.96 (12.49)	53.12 (11.65)	56.27 (13.63)	< 0.001
	CCI, Median (IQR)	1 (1)	1 (1)	1 (1)	1 (1)	1 (2)	1 (2)	1 (1)	< 0.001
	CCI 0, n (%)	296 (35.84)	247 (39.9)	243 (44.42)	187 (32.47)	194 (30.6)	298 (37.72)	1465 (36.7)	< 0.001
	CCI 1, n (%)	377 (45.64)	254 (41.03)	213 (38.94)	248 (43.06)	245 (38.64)	245 (31.01)	1582 (39.63)	< 0.001
	CCI ≥ 2, n (%)	153 (18.52)	118 (19.06)	91 (16.64)	141 (24.48)	195 (30.76)	247 (31.27)	945 (23.67)	< 0.001
	LOHS, Median IQR	17 (19)	15 (18)	15 (18)	13 (15)	14 (16)	14 (18)	15 (17)	< 0.001
	IHM, n (%)	18 (2.18)	14 (2.26)	8 (1.46)	5 (0.87)	7 (1.1)	11 (1.39)	63 (1.58)	0.237
MAJOR)	N	608	376	282	248	230	275	2019	< 0.001
	Male, n (%)	386 (63.49)	237 (63.03)	175 (62.06)	175 (70.56)	158 (68.70)	213 (77.45)	1344 (66.57)	< 0.001
	Female, n (%)	222 (36.51)	139 (36.97)	107 (37.94)	73 (29.44)	72 (31.30)	62 (22.55)	675 (33.43)	< 0.001
	Age, Mean (SD)	67.42 (13.85)	64.72 (15.53)	63.29 (16.3)	58.8 (15.73)	57.02 (13.74)	56.46 (12.13)	62.6 (15.15)	< 0.001
	CCI, Median (IQR)	1 (1)	1 (1)	1 (2)	1 (1)	1 (1)	1 (1)	1 (1)	0.007
	CCI 0, n (%)	145 (23.85)	85 (22.61)	73 (25.89)	45 (18.15)	45 (19.57)	53 (19.27)	446 (22.09)	0.163
	CCI 1, n (%)	267 (43.91)	176 (46.81)	108 (38.3)	112 (45.16)	96 (41.74)	98 (35.64)	857 (42.45)	0.043
	CCI ≥ 2, n (%)	196 (32.24)	115 (30.59)	101 (35.82)	91 (36.69)	89 (38.7)	124 (45.09)	716 (35.46)	0.002
	LOHS, Median (IQR)	18 (22)	17 (23)	18 (20)	21 (25)	21 (22)	17 (23)	18 (23)	0.499
	IHM, n (%)	58 (9.54)	29 (7.71)	26 (9.22)	22 (8.87)	16 (6.96)	22 (8)	173 (8.57)	0.829

*n* Number of procedures, *CCI* Charlson Comorbidity Index, *IQR* Inter Quartile Range, *LOHS* Length of hospital stay, *IHM* In-hospital mortality

Using the age-adjusted Poisson regression model, we found that the incidence of minor LEA for the period 2001–2019 was 3.01-fold higher among men with T1DM than among women with T1DM (IRR 3.01; 95% CI 2.64–3.36).

The mean age for patients with T1DM who underwent a minor LEA decreased significantly from 62.38 years of age in 2001–3 to 53.12 years of age in 2017–19 (p < 0.001). Overall, comorbidity measured with CCI, increased significantly over time (p < 0.001). Specifically, a significant increment was observed in the prevalence of infection, (19.52% in 2001–6 vs. 23.31% in 2013–19; p < 0.001), peripheral neuropathy, (8.93% in 2001–6 vs. 28.93% in 2013–19; p < 0.001), ischemic heart disease (3.53% in 2001–6 vs. 7.3% in 2013–19; p < 0.001), chronic kidney disease (15.02% in 2001–6 vs. 32.23% in 2013–19; p < 0.001), lipid metabolism disorder (11.63% in 2001–6 vs. 30.48% in 2013–19; p < 0.001) and liver disease, (2.28% in 2001–6 vs. 4.28% in 2013–19; p = 0.002). However, the prevalence of PAD, (76.68% in 2001–6 vs. 50.91% in 2013–19; p < 0.001) and hypertension decreased (30.93% in 2001–6 vs. 30.48% in 2013–19; p = 0.013) (Table 2).

**Table 2:** Comorbidities among patients with T1DM who had a non-traumatic lower extremity amputation (Spain 2001–2019)

		2001–06	2007–12	2013–19	Trend
MINOR	N	1445	1123	1424	0.008
	Peripheral arterial disease, n (%)	1108 (76.68)	767 (68.3)	725 (50.91)	< 0.001
	Infection, n (%)	282 (19.52)	329 (29.3)	332 (23.31)	< 0.001
	Peripheral neuropathy, n (%)	129 (8.93)	234 (20.84)	412 (28.93)	< 0.001
	Ischemic Heart Disease, n (%)	51 (3.53)	49 (4.36)	104 (7.3)	< 0.001
	Chronic Kidney Disease, n (%)	217 (15.02)	239 (21.28)	459 (32.23)	< 0.001
	Hypertension, n (%)	447 (30.93)	399 (35.53)	434 (30.48)	0.013
	Stroke, n (%)	56 (3.88)	26 (2.32)	45 (3.16)	0.082
	Heart failure, n (%)	161 (11.14)	125 (11.13)	187 (13.13)	0.175
	Lipid metabolism disorders, n (%)	168 (11.63)	240 (21.37)	434 (30.48)	< 0.001
	Liver disease, n (%)	33 (2.28)	52 (4.63)	61 (4.28)	0.002
	Dementia, n (%)	8 (0.55)	1 (0.09)	8 (0.56)	0.124
	Chronic pulmonary disease, n (%)	79 (5.47)	44 (3.92)	79 (5.55)	0.119
	Connective tissue disorder, n (%)	9 (0.62)	5 (0.45)	12 (0.84)	0.458
	Peptic ulcer, n (%)	18 (1.25)	10 (0.9)	12 (0.84)	< 0.352
	Cancer or metastatic cancer, n (%)	6 (0.42)	9 (0.8)	13 (0.91)	0.250
	Human immunodeficiency virus, n (%)	2 (0.14)	1 (0.09)	4 (0.28)	0.473
MAJOR	N	984	530	505	< 0.001
	Peripheral arterial disease, n (%)	845 (85.87)	420 (79.25)	309 (61.19)	< 0.001
	Infection, n (%)	126 (12.8)	113 (21.32)	91 (18.02)	< 0.001
	Peripheral neuropathy, n (%)	83 (8.43)	87 (16.42)	138 (27.33)	< 0.001
	Ischemic Heart Disease, n (%)	89 (9.04)	49 (9.25)	60 (11.88)	0.193
	Chronic Kidney Disease, n (%)	162 (16.46)	155 (29.25)	200 (39.6)	< 0.001
	Hypertension, n (%)	333 (33.84)	189 (35.66)	142 (28.12)	0.024
	Stroke, n (%)	93 (9.45)	39 (7.36)	38 (7.52)	0.265
	Heart failure, n (%)	181 (18.39)	66 (12.45)	103 (20.4)	0.002
	Lipid metabolism disorders, n (%)	109 (11.08)	102 (19.25)	144 (28.51)	< 0.001
	Liver disease, n (%)	34 (3.46)	13 (2.45)	28 (5.54)	0.026
	Dementia, n (%)	39 (3.96)	21 (3.96)	14 (2.78)	0.220
	Chronic pulmonary disease, n (%)	85 (8.64)	31 (5.85)	32 (6.34)	0.085
	Connective tissue disorder, n (%)	0 (0.00)	3 (0.57)	3 (0.59)	0.057
	Peptic ulcer, n (%)	12 (1.22)	8 (1.51)	3 (0.59)	0.362
	Cancer or metastatic cancer, n (%)	10 (1.02)	12 (2.26)	4 (0.79)	0.063
	Human immunodeficiency virus, n (%)	2 (0.2)	0 (0)	2 (0.4)	0.358

n: Number of procedures

Regarding in-hospital outcomes, as can been seen in Table 1, the median LOHS decreased significantly over time (17 days in 2001–3 vs. 14 days in 2017–19; p < 0.001). The IHM was 1.58% throughout the study period, with no significant change over time.

### Trends in incidence, clinical characteristics, and hospital outcomes for patients who underwent a major LEA procedure

The joinpoint analysis showed that among patients with T1DM who underwent major LEA procedures, the sex- and age-adjusted major LEA incidence rate decreased by 13.39% per year from 2001 to 2010 (Fig. 1D) and remained stable through 2019. In women with T1DM, the incidence decreased by 9.91% per year over the entire study period (Fig. 1E). In men, it decreased by 16.45% per year from 2001 to 2007 and then decreased by 4.87% per year from 2007 to 2017, although it increased, albeit not significantly (26.53% per year), from 2017 to 2019 (Fig. 1F).

As seen for minor LEAs, there was a significant increase in the proportion of men over time, from 63.49% for the first time period analysed to 77.45% for the last (Table 1).

The age-adjusted Poisson regression model showed that from 2001 to 2019, the incidence of major LEA was 1.85-fold higher among men with T1DM than among women with T1DM (IRR 1.85; 95% CI 1.31–2.38).

From 2001 to 2019, the mean age of patients with T1DM who underwent a major LEA decreased significantly (67.42 years of age vs. 56.46 years of age, p < 0.001). The distribution according to the median CCI showed a significant increase in patients with T1DM (p < 0.001). Patients with T1DM were characterized by an increased prevalence of infection, (12.8% in 2001–6 vs. 18.02% in 2013–19; p < 0.001), peripheral neuropathy, (8.43% in 2001–6 vs. 27.33% in 2013–19; p < 0.001), chronic kidney disease (16.46% in 2001–6 vs. 39.6% in 2013–19; p < 0.001), heart failure (18.39% in 2001–6 vs. 20.4% in 2013–19; p = 0.002), and lipid metabolism disorders (11.08% in 2001–6 vs. 28.51% in 2013–19; p < 0.001) (Table 2). However, the prevalence of PAD (85.87% in 2001–6 vs. 61.19% in 2013–19; p < 0.001) and hypertension (33.84% in 2001–6 vs. 28.125 in 2013–19; p = 0.024) decreased over time (Table 2).

Regarding in-hospital outcomes (Table 1), no significant changes were found over time for LOHS or IHM. Overall, median LOHS was 18 days, and crude IHM was 8.57%.

### In-hospital mortality and predictors thereof after nontraumatic LEA

As can been seen in Table 3, after minor and major LEA, crude IHM was higher in older patients, women, and those with more concomitant conditions according to the CCI. After major LEA, IHM was highest when the hospital admission occurred in the 2001–3 period (9.54% in 2001–03 vs. 8% in 2017–19; p < 0.005).

**Table 3:** In-hospital mortality among patients with T1DM who underwent minor or major non-traumatic lower extremity amputations

		IN HOSPITAL MORTALITY	
		Minor LEA	Major LEA
Age groups (Years)^a,b,^	18–49	11 (0.81)	31 (6.65)
	50–59	10 (0.92)	24 (6.15)
	60–69	10 (1.27)	31 (7.95)
	70–79	17 (3.13)	48 (10.15)
	80 +	15 (6.91)	39 (13)
Sex ^a,b^	Male	41 (1.36)	102 (7.59)
	Female	22 (2.28)	71 (10.52)
Charlson Comorbidity Index Median (IQR)^a,b^	Dead	2 (1)	2 (1)
	Alive	1 (1)	1 (1)
Peripheral arterial disease, n (%)^a,b^	No	13 (0.93)	27 (6.07)
	Yes	50 (1.92)	146 (9.28)
Infection, n (%)	No	47 (1.54)	143 (8.47)
	Yes	16 (1.70)	30 (9.09)
Peripheral neuropathy, n (%)	No	48 (1.49)	145 (8.47)
	Yes	15 (1.93)	28 (9.1)
Ischemic Heart Disease, n (%)^a,b^	No	48 (1.27)	137 (7.52)
	Yes	15 (7.35)	36 (18.18)
Chronic Kidney Disease, n (%)^a,b^	No	35 (1.14)	114 (7.59)
	Yes	28 (3.06)	59 (11.41)
Hypertension, n (%)	No	49 (1.81)	121 (8.93)
	Yes	14 (1.09)	52 (7.83)
Stroke, n (%)^b^	No	60 (1.55)	151 (8.17)
	Yes	3 (2.36)	22 (12.94)
Heart failure, n (%)^a^	No	43 (1.22)	136 (8.15)
	Yes	20 (4.23)	37 (10.57)
Lipid metabolism disorders, n (%)	No	53 (1.68)	147 (8.83)
	Yes	10 (1.19)	26 (7.32)
Liver disease, n (%)	No	59 (1.53)	163 (8.38)
	Yes	4 (2.74)	10 (13.33)
Dementia, n (%) ^b^	No	63 (1.58)	159 (8.15)
	Yes	0 (0)	14 (20.9)
Chronic pulmonary disease, n (%)	No	59 (1.56)	154 (8.23)
	Yes	4 (1.98)	19 (12.84)
Connective tissue disorder, n (%)	No	62 (1.57)	172 (8.54)
	Yes	1 (3.85)	1 (16.67)
Peptic ulcer, n (%)	No	63 (1.59)	169 (8.47)
	Yes	0 (0)	4 (17.39)
Cancer, Metastatic cancer	No	62 (1.56)	171(8.58)
	Yes	1 (3.57)	2 (7.69)
Human immunodeficiency virus, n (%)	No	63 (1.58)	173 (8.59)
	Yes	0 (0)	0 (0)
Year^b^	2001–03	18 (2.18)	58 (9.54)
	2004–06	14 (2.26)	29 (7.71)
	2007–09	8 (1.46)	26 (9.22)
	2010–12	5 (0.87)	22 (8.87)
	2013–15	7 (1.1)	16 (6.96)
	2017–19	11 (1.39)	22 (8)

*n* Number of procedures

^a^Significant association of the study variable with in-hospital mortality among patients with T1DM after minor non-traumatic lower extremity amputation.

^b^Significant association of the study variable with in-hospital mortality among patients with T1DM after major non-traumatic lower extremity amputation

For minor and major LEA, the IHM was significantly higher among those who suffered PAD, ischemic heart disease, chronic kidney disease and heart failure. Stroke and dementia were associated to increased IHM only after major LEA.

As can be seen in Table 4, after multivariable adjustment, the risk of dying in hospital among patients who underwent minor and major LEA increased with age and the presence of PAD, ischemic heart disease or chronic kidney disease. Higher IHM was associated with female sex (OR 1.37; 95% CI 1.01–1.84) and dementia only after major LEA. Whereas, heart failure increased the risk only for those who underwent a minor LEA.

**Table 4:** Predictors of in-hospital-mortality among T1DM subjects with minor or major non-traumatic lower extremity amputations

		Minor LEA OR (95% CI)	Major LEA OR (95% CI)
Age groups (Years)^,^	18–49	Reference	Reference
	50–59	1.08 (0.45–2.6)	0.87 (0.49–1.53)
	60–69	1.52 (0.63–3.65)	1.15 (0.67–1.97)
	70–79	3.28 (1.44–7.46)	1.44 (0.86–2.41)
	80 +	9.18 (3.99–21.13)	1.77 (1.09–2.97)
Sex	Male	Reference	Reference
	Female	1.39 (0.8–2.39)	1.37 (1.01–1.84)
Peripheral arterial disease	Yes	2.07 (1.16–3.49)	1.70 (1.08–2.89)
Ischemic Heart Disease	Yes	3.85 (2.02–7.37)	2.52 (1.67–3.81)
Chronic Kidney Disease	Yes	3.32 (1.9–5.81)	1.98 (1.37–2.86)
Stroke	Yes	NIM	1.28 (0.91–2.12)
Heart failure	Yes	2.22 (1.25–3.95)	NIM
Dementia	Yes	NIM	2.59 (1.34–5.02)
Year	2001–03	Reference	Reference
	2004–06	1.14 (0.55–2.36)	0.82 (0.51–1.32)
	2007–09	0.86 (0.36–2.05)	0.97 (0.59–1.6)
	2010–12	0.48 (0.17–1.34)	1.02 (0.6–1.74)
	2013–15	0.53 (0.21–1.33)	0.82 (0.45–1.5)
	2017–19	0.74 (0.33–1.66)	0.95 (0.55–1.64)

OR. Odds Ratio adjusted with logistic regression by variables shown in the table.

*CI* Confidence intervals, *NIM* Not included in the model

Finally, the time trend analysis showed no change in IHM from 2001–3 to 2017–19 in T1DM patients who underwent minor and major LEAs.

## Discussion

This nationwide registry and population-based observational study showed a decline in hospital admissions for minor and major LEAs in patients with T1DM from 2001 to 2009–10, with no subsequent changes. However, from 2017 to 2019, the incidence of major LEA seems to have increased among men with T1DM. The incidence rates of minor and major LEA in men with T1DM were higher than in women with T1DM. Female sex is a predictor of IHM in patients with T1DM following major LEA. In the fully adjusted model, women with T1DM had a 37% higher adjusted risk of dying following major LEA than men with T1DM.

According to our database, trends in incidence decreased significantly between 2001 and 2009 for minor LEA (9.55% per year) and between 2001 and 2010 for major LEA (13.39% per year) and subsequently remained stable for major LEA. However, for minor LEA the incidence increased, albeit not significantly, between 2010 and 2019. These findings have been reported elsewhere in Europe [4, 7]. In a population-based cohort study in Sweden, Hallstrom et al. [4] found that the frequency of amputation in patients with T1DM decreased between 1998 and 2019 and concluded that these changes are possibly related to an increased focus on risk factor management, advanced treatments to optimise glycaemic control, and further enhancements in the armamentarium of multidisciplinary diabetes foot clinics, including an increased use of invasive arterial reperfusion.

Although decreasing rates of LEA may reflect improved care, they may also reflect delayed presentation and early death in patients with foot ulceration or ischemia [19]. Consistent with these findings, the cross-sectional study of patients with T1DM and T2DM by Geiss et al. [8] performed in the USA found an increase in diabetes-related LEA rates between 2009 and 2015. This increment was driven by a 62% increase in the rate of minor amputations (from 2.03 [95% CI 1.83–2.22] to 3.29 [95% CI 3.01–3.57], p < 0.001) and a smaller, but also statistically significant, 29% increase in major LEAs (from 1.04 [95% CI 0.94–1.13] to 1.34 [95% CI 1.22–1.45]). Furthermore, these increases were more pronounced among the young and middle-aged groups (age 18–64 years), raising concerns that preventive efforts were not reaching certain subgroups of people with diabetes who may have different disease characteristics and needs [8]. It has been suggested that young people with diabetes have less motivation for diabetes self-care than for other life priorities and are also more vulnerable to diabetes-related distress and other mental health problems, which adversely affect disease control and increase the risk of complications [20]. Regarding this point, the non-significant increase observed in the incidence of LEA in men with T1DM in recent years should be closely monitored to identify a possible change in trend.

As we expected, our results show a high ratio of minor LEA to major LEA among patients with T1DM, possibly because of more conservative limb salvage procedures and increasingly more aggressive treatment of peripheral arterial disease in this population [21].

The results of the present study are in line with those found in the literature, namely, an increasing male predominance in minor and major LEAs [2, 4, 6, 21]. Similar sex differences were reported in a study conducted using data from the IBM Market-Scan research database among patients with T1DM and T2DM [22]. Male sex appears to be a greater risk factor for LEA than age, probably because of the differential sex distribution of risk factors, such as smoking, which were not measured. In addition, the protective role of oestrogen could lead to differences in immune system function between males and females. Finally, the biological factors of diabetic foot ulcer, peripheral vascular disease, coronary artery disease, and peripheral neuropathy might account for the significant sex difference in the amputation rates [23, 24].

While the results of the present study indicate that hypertension decreased over time, the frequency of chronic kidney disease and lipid metabolism disorders increased in patients who underwent minor and major LEA. The association between LEA and person-level risk factors in individuals with T1DM has been studied elsewhere [2, 4, 22, 25]. Hallstrom et al. [4] recently described cardiovascular comorbidities, renal dysfunction, increased HbA1c, hypertension, and smoking to be associated with an increased risk of amputation in patients with T1DM.

IHM rates for minor and major LEAs did not change significantly between 2001 and 2019. The current results reinforce those previously found in Spain for the period between 2001 and 2012 [6]. This lack of improvement over time should be investigated to identify possible explanations and implement protective measures.

During admission for major LEA, women with T1DM had a higher IHM, which remained unchanged after the multivariable regression analysis. Results on the influence of sex on IHM following LEA are contradictory. Various studies found that amputation was more frequent in men than in women [25]; however, female sex was a predictor of in-hospital mortality after LEA in a previous study in Spain [6]. Gurney et al. [26] found little difference between the sexes in terms of the adjusted risk of postoperative mortality (90 days) and concluded that female patients who undergo amputation are similar to their male counterparts in terms of the underlying risk factors for postoperative mortality.

Gender differences in glucose control in T1DM subjects, with women showing a worst glucose control possibly due to pathophysiologic differences rather than the care provided, could also contribute to the differences observed [27].

It has been repeatedly demonstrated that the presence of diabetes increases the risk burden for cardiovascular complications among females. The mechanisms involved in the excess risk for vascular complications due to diabetes in females are the object of discussion and investigation and include hormonal factors, genetic predisposition linked to sex dimorphism, and sex-related inequalities in treatment or socio-economic status, which individually or together seem to contribute to the higher incidence in females [28, 29]. Indeed, in Spain, a sex gap has been reported in the impact of diabetes on IHM after major cardiovascular events [30]. The multifactorial causes for excess risk among women with T1DM require further investigation.

In our investigation, as reported in previous studies, the comorbidities that were significant predictors for death after LEA included PAD, ischemic heart disease or chronic kidney disease [31–34]. Morbach et al., found that the long-term survival after LEA is poor, especially among people with diabetes with concomitant PAD, renal insufficiency or the combination of both [31]. Diabetic patients with PAD, compared with those without PAD, have a higher risk of cardiovascular and all-cause mortality [32].

Interestingly, in our study neuropathy was not significantly associated with death as reported before [31]. Even more, Cascini et al. reported a protective effect of neuropathy on mortality after major LEA. We agree with these authors that a possible explanation is a differential under-reporting in hospital charts of peripheral neuropathy [33]. Indeed, the diagnosis of neuropathy is a complex process and requires expertise that is not always available [33].

The strength of our study lies in its large sample size (6,011 nontraumatic LEA procedures in patients with T1DM), coverage of an entire country (> 95% of all hospital admissions), and standardized methodology (extensively used for research in Spain, combined with the reliability of diabetes and LEA coding in the RAE-CMBD) [5, 6, 30]. Nevertheless, our study is subject to a series of limitations. Our data were obtained from an administrative database supported by the information that physicians recorded in the discharge report; therefore, the database lacks information on clinical characteristics, glycaemic control, medical treatments, and time with T1DM. Another limitation is that anonymity precludes the extraction of specific data that may affect the results (i.e., people who moved from one hospital to another could appear twice). However, Buckley et al. [35] detected high levels of agreement between hospital discharge data and medical records for LEA and diabetes and suggested that hospital discharge data are sufficiently reliable for monitoring trends in LEA in people with diabetes. In our opinion, the strengths of this study and its uniqueness clearly outweigh its limitations.

## Conclusions

In conclusion, our data show a decrease in the incidence of minor and major LEA in men and women with T1DM and a slight but not significant increase in the incidence of major LEA in men with T1DM during the last years of the study. This possible change in the time trends must be closely followed in the future. The incidence of minor and major LEA was higher in men than in women. Female sex is a predictor of IHM in patients with T1DM following major LEA. As the study was based on administrative data, we lack relevant clinical data, thus making it necessary to take into account the effect of residual confounding. Our results should be interpreted with caution and confirmed using data from clinical studies. However, our results should be the starting point for future investigations that can confirm or reject our conclusions using more extensive and precise clinical data.

## Supplementary Information

**Additional file 1: Table S1.** International Classification of Disease, 9th edition (ICD-9-CM) and 10th edition, (ICD-10) codes for the clinical diagnoses and procedures used in this investigation.

## Data Availability

According to the contract signed with the Spanish Ministry of Health and Social Services, which provided access to the databases from the Spanish National Hospital Database (*Conjunto Mínimo Basico de Datos*; CMBD), we cannot share the databases with any other investigator, and we have to destroy the databases once the investigation has concluded. Consequently, we cannot upload the databases to any public repository. However, any investigator can apply for access to the databases by filling out the questionnaire available at http://www.mscbs.gob.es/estadEstudios/estadisticas/estadisticas/estMinisterio/SolicitudCMBDdocs/Formulario_Peticion_Datos_CMBD.pdf. (Accessed on 9 September 2021) All other relevant data are included in the paper.
