# Supplementary material for: Time trends (2001–2019) and sex differences in incidence and in-hospital mortality after lower extremity amputations among patients with type 1 diabetes in Spain
Source: Cardiovasc Diabetol. 2022 May 3;21:65. doi: 10.1186/s12933-022-01502-y (PMC9066863; doi:10.1186/s12933-022-01502-y)
Supplement: Supplementary file 1 — Additional file 1: Table S1. International Classification of Disease, 9th edition (ICD-9-CM) and 10th edition, (ICD-10) codes for the clinical diagnoses and procedures used in this investigation. [file 12933_2022_1502_MOESM1_ESM.docx]

**Supplementary Table 1. International Classification of Disease, 9^th^ edition (ICD-9-CM) and 10th edition, (ICD-10) codes for the clinical diagnoses and procedures used in this investigation.**

|  | **ICD-9 code** | **ICD-10 code** |
| --- | --- | --- |
| **Type 1 diabetes** | 250.x1; 250.x3. | E10.xxx |
| **Type 2 diabetes (exclusion)** | 250.x0; 250.x2 | E11.xxx |
| **Level of NLEA** |  |  |
| Toe | 84.11 | 0Y6P – 0Y6Y |
| Foot | 84.12, 84.13 | 0Y6M – 0Y6N |
|  |  |  |
| Below the knee amputation (BKA) | 84.14, 84.15, 84.16 | 0Y6F, 0Y6G, 0Y6H, 0YJ |
| Above the knee amputation (AKA) | 84.17, 84.18, 84.19 | 0Y62, 0Y63, 0Y64, 0Y67, 0Y68, 0Y6C, 0Y6D |
| Traumatic amputations (exclusion) | 895, 896, 897 | S78, S88, S98 |
| Peripheral arterial disease | 785.4, 440.2x-440.9x, 440.3x, 440.4x, *443.81,* 443.89, 443.9, 444.22, 445.02 | I96, I70.2xx- I70.9xx, I73.89, I73.9, I74.3  I75.029 |
| Infection | 040.0, 680.6, 681.10, 682.5, 682.6, 682.7, 728.86, 730.05, 730.06, 730.07, 730.15, 730.16, 730.17, 730.25, 730.26, 730.27, 730.85, 730.86, 730.87, 730.95, 730.96, 730.97 | A48.0, L02.429, L02.611, L02.612, L02.619, L03.039, M72.6, M86.05x, M86.06x, M86.07x, M86.15x, M86.16x, M86.17x, M86.25x, M86.26x, M86.27x, M86.35x, M86.36x, M86.37x,M86.45x, M86.46x, M86.47x, M86.55x, M86.56x, M86.57x, M86.65x, M86.66x, M86.67x, M86.8X5, M86.8X6, M86.8X7 |
| Peripheral neuropathy | 355, 357.2, 536.3, 713.5 | E10.40, E10.41, E10.42, E10.43, E10.610 |
| Ischemic heart disease | 410-414 | I20-I25 |
| Chronic kidney disease | 585 | N18 |
| Hypertension | 401.1, 401.9, 642.0 | I10, I16.6 |
| Stroke | 362.34, 430.x–438.x | I60-I66 |
| Heart failure | 398.91,402.01,402.11,402.91,404.01, 404.03,404.11,404.13,404.91,404.93,425.4–425.9, 428.x | I09.9, I11.0, I13.0, I13.2, I25.5, I42.0, I42.5–I42.9, I43.x, I50.x, P29.0 |
| Lipid metabolism disease | 272,0-272.4, 272.8, 272.9 | E78.0X-E78.5 |
| Liver disease | 070.22, 070.23, 070.32, 070.33, 070.44, 070.54, 070.6, 070.9, 570.x, 571.x, 573.3, 573.4, 573.8, 573.9, V42.7, 456.0–456.2, 572.2–572.8 | B18.x, K70.0 - K70.3, K70.9, K71.3 - K71.5, K71.7, K73.x, K74.x, K76.0, K76.2 - K76.4, K76.8, K76.9, Z94.4, I85.0, I85.9, I86.4, I98.2, K70.4, K71.1, K72.1, K72.9, K76.5, K76.6, K76.7 |
| Dementia | 290.x, 294.1, 331.2 | F00.x–F03.x, F05.1, G30.x, G31.1 |
| Chronic pulmonary disease | 416.8, 416.9, 490.x–505.x, 506.4, 508.1, 508.8 | I27.8, I27.9, J40.x–J47.x, J60.x–J67.x, J68.4, J70.1, J70.3 |
| Connective tissue disorder | 710.0, 710.1, 710.4, 714.0, 714.1, 714.2, 714.81, 725.x | M05.x, M06.x, M31.5, M32.x, M33.x, M34.x, M35.1, M35.3, M36.0 |
| Peptic ulcer | 531.x, 532.x, 533.x, 534.x | K25.x, K26.x, K27.x, K28.x |
| Cancer or metastatic cancer | 140.x–172.x, 174.x–195.8, 200.x–208.x, 238.6, 196.x–199.x | C00.x - C26.x, C30.x - C34.x, C37.x - C41.x, C43.x, C45.x - C58.x, C60.x - C76.x, C81.x - C85.x, C88.x, C90.x - C97.x, C77.x - C80.x |
| Human immunodeficiency virus | 042.x – 044.x | B20.x - B22.x, B24.x |

Codes obtained from references #15 and #16
